# The Assessment of Changes in the Fatty Acid Profile and Dietary Indicators Depending on the Storage Conditions of Goose Meat

**DOI:** 10.3390/molecules26175122

**Published:** 2021-08-24

**Authors:** Agnieszka Orkusz, Wioletta Wolańska, Urszula Krajinska

**Affiliations:** 1 Department of Biotechnology and Food Analysis, Wroclaw University of Economics and Business, 53-345 Wroclaw, Poland; 2 Department of Forecasts and Economic Analysis, Wroclaw University of Economics and Business, 53-345 Wroclaw, Poland; wioletta.wolanska@ue.wroc.pl; 3 St George’s University Hospitals NHS Foundation Trust, Blackshaw Road Tooting, London SW17 0QT, UK; urszula.krajinska@kcl.ac.uk

**Keywords:** human nutrition, fatty acids, goose, modified atmosphere, vacuum

## Abstract

The deterioration of food quality due to lipid oxidation is a serious problem in the food sector. Oxidation reactions adversely affect the physicochemical properties of food, worsening its quality. Lipid oxidation products are formed during the production, processing, and storage of food products. In the human diet, the sources of lipid oxidation products are all fat-containing products, including goose meat with a high content of polyunsaturated fatty acids. This study aims at comparing the fatty acid profile of goose breast muscle lipids depending on the storage conditions: type of atmosphere, temperature, and storage time. Three-way variance analysis was used to evaluate changes in the fatty acids profile occurring in goose meat. The health aspect of fatty acid oxidation of goose meat is also discussed. In general, the fatty acid composition changed significantly during storage in the meat packed in the high-oxygen modified atmosphere at different temperatures (1 °C and 4 °C). Higher temperature led to a higher degree of lipid oxidation and nutrient loss. During the storage of samples in vacuum, no changes in the fatty acid content and dietary indices were found, regardless of the storage temperature, which indicates that the anaerobic atmosphere ensured the oxidative stability of goose meat during 11 days of refrigerated storage.

## 1. Introduction

Due to the growing awareness of diseases resulting from unhealthy diets and the dietary recommendations made by the international organizations concerned with nutrition (Food and Agriculture Organization of the United Nations, FAO; World Health Organization, WHO; and World Cancer Research Fund, WCRF), a lot of recent research has been focused on the characterization of fatty acid profiles, starting with plant products and novel foods [1,2,3,4]; however, consumers are looking for meat low in lipids and high in protein. For these reasons, consumers both in the European Union and worldwide prefer poultry meat [5]. Consumption of poultry meat is expected to increase globally to 145 Mt by 2029. Among all the additional meat consumed over the next decade, poultry is expected to account for 50% [6].

Poultry meat has high nutritional and dietary value due to low lipid content (breast meat: 1.48–4.25%), and high content of protein (breast meat: 19.85–24.31%), minerals (Fe, Zn, Mg, Na, K, P, Ca), and vitamins B (Table 1).

Poultry meat is a source of unsaturated fatty acids (mono and polyunsaturated), which in goose meat constitute almost 70% of the total fatty acids content [8,9].

The following acids are of particular importance in the human diet: linolenic (C 18:3 n-3) and linoleic (C 18:2 n-6), which are the precursors of polyunsaturated acids from the n-3 and n-6 family, respectively. The human body cannot synthesize essential fatty acids, so they must be supplied with food. It is also noted that the imbalance caused by excessive consumption of n-6/n-3 fatty acids negatively affects the lipid profile, and increases the risk of oxidative stress and obesity development [10]. Polyunsaturated acids, followed by monounsaturated fatty acids (MUFA), are particularly susceptible to oxidation during meat storage [11,12]. Saturated fatty acids are resistant to oxidation [13].

Oxidation leads to a deterioration of sensory quality and widely affects the product’s nutritional value and health safety [14,15]. Lipid peroxidation is believed to be one of the possible pathways for the development of the cancer process [12]. Reactive oxygen species (ROS) can damage lipids, the oxidized forms of which become involved in the chain of free radical reactions and increase the potential damage to a lot of body structures. The preferred site of the attack by ROS is the site of the PUFA carbon chain between the double bonds. Peroxidation of phospholipids located in the cell membrane disrupts the exchange of electrolytes and water between the interior of the cell and the external environment [12]. This weakens cell function, promotes dehydration, and can lead to premature death (necrosis). This process can create favourable conditions for the development of numerous disorders.

It is worth pointing out that moderate oxidative changes are not entirely undesirable. To some extent, they are responsible for forming the palatability of meat and its products. In the oxidation process, unsaturated fatty acids can produce a variety of products, such as ketones, aldehydes, alcohols, and acids, which in low concentrations give the products the right flavor [16].

Factors that slow down the oxidation reaction include reduced temperature and oxygen pressure and the inert gas environment [17].

The amount of fatty acid oxidation products can be reduced by creating appropriate food storage conditions. Therefore, packaging in a modified atmosphere (vacuum and gas atmosphere) is used for the storage of meat.

The slaughter of geese is conducted seasonally, which in Poland means the period from June to early December. During this period, goose meat is offered to consumers in the form of the whole poultry and culinary items stored refrigerated in vacuum or conditions of modified atmosphere (most of the composition 80% O_2_, 20% CO_2_). An essential condition for ensuring the high quality and health safety of fresh meat stored in a modified atmosphere is to maintain a temperature of no more than 4 °C, preferably 0–2 °C. Lipid oxidation is one of the main causes of stored goose meat quality deterioration, as it is rich in unsaturated fatty acids. Therefore, it is essential to evaluate the changes in the fatty acid profile of meat resulting from its storage method.

This study aims at assessing changes in the fatty acid profile and the dietary indicators of goose breast muscle lipids depending on the storage conditions: type of atmosphere, temperature, and storage time.

The results of the research will indicate whether the nutritional value of goose meat stored in the altered atmospheres is reduced, primarily in terms of a reduction in the amount of unsaturated fatty acids in the muscles’ lipids.

## 2. Results

Based on the analysis of variance results, it was shown that for fatty acids (except for C 18:1 and UFA) and dietary indicators, the main effects were significant for all factors considered (time, atmosphere, and temperature), for the adopted significance level of *p* < 0.05. Most effects were also found to be significant for the two- and three-factor interactions. The three-way interaction effect did not occur with MUFA, C 18:1, C 20:4 n-6, or n-3. The obtained results confirm that the analyzed meat storage conditions significantly affect the content of fatty acids and the determined indicators (Table 2). For a more detailed analysis of the changes taking place in the fatty acid profile, the post hoc test was used according to the Tukey’s procedure for a significance level of *p* < 0.05.

The profile, proportions, and indices of fatty acids of the goose breast muscles packed in the protective atmospheres and stored at 1 °C and 4 °C for 11 days are presented in Table 3.

Throughout the whole time, regardless of the storage temperature, there was no change in the fatty acid content in the samples packed in vacuum (Table 3). Therefore, for the dietary indicators calculated based on the content of individual fatty acids, no significant difference was noted during the storage of goose muscles in a vacuum at 1 °C and 4 °C.

For the goose breast muscles packed in the modified atmosphere and stored at 1 °C and 4 °C, no significant changes were observed in the palmitic acid content (C 16:0), arachidonic acid (C 20:4), NV, or h/H indices until day 4 of storage. In the case of other fatty acids and the indexes calculated on their basis, some significant changes occurred on day 4 of storage, both at 1 °C and 4 °C (Table 3). This means that the storage of the breast muscles in the modified high-oxygen atmosphere at 1 °C and 4 °C causes unfavourable changes in the content of fatty acids and the dietary indicators.

The content of saturated fatty acids increased during the storage time of muscles packed in the modified atmosphere, both at 1 °C and 4 °C. At the temperature of 1 °C, the content of saturated fatty acids increased by an average of 0.37, 1.42, and 2.03 percentage points on the 4th, 7th, and 11th day, respectively, compared to the initial value. In contrast, in the muscles stored at 4 °C, an increase in SFA by 0.50, 1.49, and 3.47 percentage points was found on day 4, 7, and 11, respectively. It should be noted that while on the 4th and 7th day of storage, the differences in the SFA content of muscles stored at different temperatures were not large, the difference was significant on the 11th day. The observed upward trend in the SFA content resulted mainly from the increase in the palmitic acid. Statistically significant changes in the content of this acid, regardless of the storage temperature, were recorded on days 7 and 11. On the 7th day at 1 °C and 4 °C, the palmitic acid content increased by 0.82 and 0.83 percentage points, respectively, and on the 11th day, by 0.9 and 1.95 percentage points, respectively, compared to the initial value (Table 3).

Based on the obtained results, an increase in the MUFA content was also recorded during the storage of goose muscles in MA. At the temperature of 1 °C, the content of monounsaturated fatty acids increased by an average of 0.96, 1.34, and 2.22 percentage points, respectively, on the 4th, 7th, and 11th day, compared to the initial value. On the other hand, in the muscles stored at 4 °C, an increase in MUFA by 1.23, 1.50, and 2.67 percentage points was registered on the 4th, 7th, and 11th day of storage, respectively. The observed upward trend in the content of MUFA was caused by an increase in the content of oleic acid (C 18:1), which is the main representative of monounsaturated acids in goose muscles. According to the analysis of variance, statistically significant changes in the content of this acid, regardless of the temperature, were recorded on days 4, 7, and 11 of storage. On the 4th day at 1 °C and 4 °C, the oleic acid content increased by 1.10 and 1.15 percentage points, respectively, on the 7th day by 1.20 and 1.22 percentage points, and on the 11th day of storage, there was an increase of 1.87 and 2.06 points, compared to the initial value. It should be emphasized, as in the case of saturated fatty acids, that on days 4 and 7 of storage, the differences in the content of MUFA in the muscles stored at different temperatures were not large, while on day 11, a much higher content of MUFA was recorded in the muscles stored at 4 °C (Table 3).

Regardless of the storage temperature, the most significant changes in the content of polyunsaturated fatty acids were observed in the muscles packed in the modified atmosphere, compared to the content of SFA and MUFA. The amount of PUFA decreased during the storage time of goose muscles. At 1 °C, the polyunsaturated fatty acids content decreased on average by 1.17, 2.54, and 4.54 percentage points on the 4th, 7th, and 11th day, respectively. Meanwhile, in the muscles stored at 4 °C, a decrease in PUFA by 1.23, 2.70, and 5.78 percentage points was registered. Based on the obtained results, it was found that on the 4th, 7th, and 11th day of storage the differences in the content of polyunsaturated fatty acids in the muscles stored at 1 °C and 4 °C were significant. The observed downward trend in the PUFA content was mainly due to the decrease in the content of linoleic acid, linolenic acid, and arachidonic acid. Statistically significant changes in the content of polyunsaturated fatty acids were recorded on days 4, 7, and 11, regardless of the storage temperature. On the 4th day at 1 °C and 4 °C, the linoleic acid content decreased compared to the initial value by 0.92 and 0.90 percentage points, respectively, on the 7th day by 1.42 and 1.45, and on the 11th day by 2.66 and 3.74 percentage points, respectively. Compared to the initial value, the decrease in the content of the linolenic and arachidonic acid at the end of the storage time was by 0.45 and 1.14 percentage points at 1 °C, and by 0.46 and 1.24 percentage points at 4 °C, respectively (Table 3).

The decrease in the PUFA content in the goose muscles resulted in reducing the content of UFA, PUFA n-6, and PUFA n-3 on day 11 of muscle storage by 2.32, 3.87, and 0.67 percentage points for 1 °C and by 3.11, 5.11, and 0.67 percentage points for 4 °C, respectively. At the same time, an increase in the ratio of the sum of n-6/n-3 polyunsaturated fatty acids was observed during storage. At 1 °C, there was an increase in n-6/n-3 by an average of 1.38, 1.97, and 2.31 percentage points on days 4, 7, and 11, respectively, compared to the initial value. On the other hand, in the muscles stored at 4 °C, an increase of n-6/n-3 by 0.87, 1.38, and 1.54 percentage points was found on days 4, 7, and 11. The decrease in PUFA, combined with an increase in the content of SFA in muscles stored in MA, resulted in the reduction of the PUFA/SFA ratio. At the temperature of 1 °C, the PUFA/SFA value decreased by an average of 0.05, 0.12, and 0.2 percentage points on the 4th, 7th, and 11th day. In the meat stored at 4 °C, the losses of PUFA/SFA amounted to 0.05, 0.13, and 0.26 percentage points, respectively (Table 3).

According to the analysis of variance, a statistically significant increase in the athero-genic, thrombogenic, and saturation indexes took place on the 4th, 7th, and 11th storage day, regardless of the temperature. On the 4th day at 1 °C and 4 °C, the atherogenic index increased by 0.01 percentage points, compared to the initial value, by 0.03 on the 7th day, and by 0.04 and 0.06 percentage points on the 11th day, respectively. The amount of the thrombogenic index and saturation at the end of the storage time, compared to the initial value, increased, respectively, at 1 °C by 0.10 and 0.04 percentage points, and at 4 °C by 0.15 and 0.07 percentage points. While on days 4 and 7 the differences in changes in the values of atherogenic, thrombogenic, and saturation indexes at the storage temperature of 1 °C and 4 °C were not large, on day 11 they were very significant.

For the samples packed in MA and stored both at 1 °C and 4 °C, negative changes were observed, consisting of a reduction in the proportion of acids with hypo- and hypercholesterolemic effects. As shown by the analysis of variance, a statistically significant decrease in the h/H ratio was recorded from the 7th storage day, regardless of the temperature. On the 11th day at 1 °C and 4 °C, the losses in the h/H ratio were 0.21 and 0.34 percentage points, respectively (Table 3).

## 3. Discussion

The data concerning fresh raw breast generally agree with the corresponding values reported by [18,19] for intensively reared 10-week-old White Kołuda^®^ goose broilers (W31)—basic commercial crosses for meat production in Poland—and [20] for 17-week-old White Kołuda^®^ (W31) goose raised in the semi-intensive system and fattened by oat ad libitum. The main fatty acids in the unpacked goose meat included palmitic acid (saturates), oleic acid (monounsaturates), and linoleic acid (polyunsaturates). Palmitic acid belongs to the group of acids which are believed to harm the human body, especially by stimulating blood cholesterol, especially its LDL fraction [8,21]. It is considered that palmitic acid is associated with disadvantageous cardiovascular events [22]. Replacing fats rich in saturated fatty acids in the diet with polyunsaturated fatty acids from the n-3 and n-6 family is associated with a reduction, by approximately 30%, in the risk of developing cardiovascular disease [23] and has a significant impact on reducing the risk of the sudden cardiac arrest [24]. The recommended maximum amounts of saturated fatty acids in the diet of adults constitutes 5–6% of the energy of the food ration [25,26,27,28,29].

Among all unsaturated fatty acids, the highest percentage was found for oleic acid. Its positive effect on the human body has been noted many times; however, it is related to the content of n-3 and n-6 acids [30,31]. At the same time, the content of n-6 acids in the unpackaged goose breast muscle was ten times higher than in the n-3 family. It is therefore essential to diversify the diet and enrich it with products containing n-3 poly-unsaturated fatty acids.

The scope of oxidative changes in goose muscle lipids depended on the storage conditions: the atmosphere type (oxygen amount in the package), temperature, and storage time. It is well known that higher storage temperature and the presence of oxygen accelerate oxidation processes [32]. Thus, changes were expected in the samples stored at higher temperature and in the high-oxygen atmosphere. However, it was unknown how much the fatty acid profile of the samples stored in MA would change and whether the fatty acid profile of the samples stored in the oxygen-free atmosphere would change.

The type of protective atmosphere significantly differentiated the percentages of all the determined groups of fatty acids, as well as the values of all presented proportions and dietary indexes during 11-day storage of muscles at 1 °C and 4 °C (Table 3). During the storage of the samples in vacuum, no changes in the fatty acid content and dietary indexes were found, regardless of the storage temperature, which indicates that the anaerobic atmosphere ensured the oxidative stability of the goose meat during the 11 days of refrigerated storage. At the same time, there was an increase in SFA, MUFA, n-6/n-3 ratio, and a decrease in PUFA and UFA in the muscles stored in MA. The higher storage temperature of goose muscles caused more significant changes in the analyzed fatty acids and dietary indicators, with apparent differences observed on the 11th storage day.

It should be noted that the quality of the consumed fat, not its quantity, is the most important factor in the prevention of diseases, including those that are diet related [33]. The deficiency of unsaturated fatty acids, especially from the n-3 family, disrupts brain function [34,35] and adversely affects vascular functions, leading to an increased risk of damage to the endothelium of blood vessels [36,37].

Moreover, n-3 deficiency disturbs the functioning of the cardiovascular system, for example by influencing the concentration of triglycerides and the LDL-cholesterol fraction in blood serum [37]. The excess of n-6 family acids in comparison to n-3 family acids causes the pro-aggregation processes of platelets to prevail over the anti-aggregation processes, which increases the risk of atherosclerotic changes in blood vessels [38]. A diet rich in n-6 acids and low in n-3 is essential in the pathogenesis of cancer development [39].

The results of our own research indicate an increase in the intensity of lipid oxidation processes in the goose muscles stored in the presence of oxygen. Under aerobic conditions, the influence of temperature on muscle quality was also noted; it deteriorated at a higher storage temperature—at 4 °C.

Based on our research, it was found that the health-promoting quality of meat stored in MA is lower than in vacuum. During the storage of the samples in the oxygen atmosphere, an increase in the values of the AI, TI, S/P, and NV indexes and a decrease in h/H were noted. Meanwhile, for the vacuum stored samples, the indexes mentioned did not change, regardless of the storage temperature.

Therefore, it can be univocally concluded that storing the goose meat in the high oxygen atmosphere caused negative changes in the fatty acids and dietary indicators and thus decreased its nutritional value. The anaerobic atmosphere protected the meat from changes in the fatty acid profile, so its nutritional value was not reduced.

## 4. Materials and Methods

### 4.1. Meat Preparation

Geese came from the same farm. According to the fattening program for White Kołuda^®^ geese [40], birds were fed in a semi-intensive system up to the 17th week of age. Their nutritional intake up to the 14th week was mainly green forage, and cereal grain with a small addition of concentrate feeds. From the 15th to the 17th week of age, geese were fattened with whole oat grain without any limits.

The raw material was the breast muscles of the 17-week-old White Kołuda^®^ goose from industrial slaughter. The meat was individually packed in a slaughterhouse, using type R-230 Multivac wrapper in polyamide-polyethylene foil (permeability: O_2_ = 25 cm^3^/m^2^∙24h∙0.1MPa; CO_2_ = 85 cm^3^/m^2^∙24h∙0.1MPa; N_2_ = 7 cm^3^/m^2^∙24h∙0.1MPa; water vapor < 3 g/m^2^∙24h) in a vacuum (99%) and a high-oxygen modified atmosphere with a composition of 80% O_2_ and 20% CO_2_ (MA). Samples for experiments were chosen randomly. In total, 325 goose breast muscles were examined. Three hundred muscles were randomly assigned to two different atmospheres. One hundred and fifty muscles were packed in the high-oxygen modified atmosphere (75 muscles were stored at 1 °C and 75 at 4 °C) and 150 in a vacuum (75 muscles were stored at 1 °C and 75 at 4 °C). The control group consisted of 25 unpacked muscles, which were examined 24 h after the slaughter. Packed muscles refrigerated at 1 °C and 4 °C were tested on the 4th, 7th, and 11th day of storage. Fifteen portions of the breast muscles were examined in each packaging method (5 individually packed samples were tested on the 4th, 7th, and 11th day). The experiments were repeated 5 times for both atmosphere types. The determinations were performed for each of the sample in two repetitions.

### 4.2. Lipid Extraction

Lipid fraction extracts contain large amounts of non-lipid water-soluble compounds such as amino acids, sugars, and others. These substances had to be removed from the sample before starting the analysis. The Folch method was used [41] for this purpose.

### 4.3. Preparation of Fatty Acid Methyl Esters (FAMEs)

The fat extract (2 mL) was transferred into a 20 mL tube and placed in the heating block. The extract was evaporated in a stream of nitrogen at 70 °C. Then, 1 mL of 2 M KOH in 75% methanol was added and mixed. The tube was capped and heated at 70 °C for one hour. After the extract was cooled down, 1 mL of n-hexane was added (extraction of unsaponifiable substances), it was shaken by hand for 5 min, then the hexane layer was removed. Unsaponifiables extraction was repeated. In the next step, 1 mL 2 M aqueous HCl solution was added. The tube was capped and heated for 30 min in a heating block at 70 °C. After cooling it to room temperature, 1 mL of *n*-hexane and approximately 5–7 mL of saturated aqueous NaCl solution was added to the sample and shaken by hand for 5 min. The hexane extract was collected in a vial (5 mL) with anhydrous Na_2_SO_4_ (1.0 g). The extraction process was repeated. The vial was capped, mixed, and then set aside in the refrigerator for 24 h. The hexane extract was then poured into a tube (20 mL) and evaporated to dryness in a stream of nitrogen at 70 °C. After cooling the extract at room temperature, 1 mL of 0.5 M KOH solution in anhydrous methanol was added. The tube was closed with a stopper, then mixed. The sample was heated for 30 min at 70 °C. After cooling, 1 mL of 1 M HCl in anhydrous methanol was added to the extract and warmed again for 30 min at 70 °C. After cooling, 1 mL of hexane and approximately 5–7 mL of saturated water NaCl solution was added to the extract and shaken for 5 min. After separating the layers in a tube, the hexane layer was collected into a sodium sulfate vial. The extraction process was repeated. The prepared sample containing methyl esters was marked using gas chromatography.

### 4.4. Gas Chromatographic Analysis

The FA methyl esters were quantified by gas chromatography using Agilent Technologies 7890 A series gas chromatograph (Agilent Technologies Inc., St. Clara, CA, USA) equipped with a silica capillary column HP 88 J&W Scientific series—100 m × 0.25 mm × 0.20 µm film thickness and flame-ionization detector (FID) at injection volume of 1.0 μL and split ratio 1/50, respectively. Helium was used as the carrier gas at a head pressure of 2.0 mL/min constant flow. The temperatures of the detector and injector were 280 °C and 250 °C, respectively. The initial column temperature was set at 120 °C for 1 min, then raised to 175 °C at 10 °C/min and held for 10 min. Then, it was increased to 210 °C at 5 °C/min, held for 5 min, and finally raised to 230 °C at a rate of 5 °C/min. Fatty acids were presented as a percentage of the total amount of the methyl esters.

Among the determined fatty acids, the following groups were selected: saturated fatty acids, monounsaturated fatty acids, polyunsaturated fatty acids, unsaturated fatty acids, polyunsaturated n-6, and polyunsaturated n-3. The proportions of PUFA/SFA and n-6/n-3 fatty acids were calculated from the content of individual fatty acids.

### 4.5. Dietary Indicators

Atherogenic indexes (AI), thrombogenic index (TI), saturation index (S/P) [42], lipid nutritional value (NV) [43], and hypocholesterolemic/hypercholesterolemic ratio (h/H) [44,45] were calculated according to the following equations:

$$\mathrm{AI}=\frac{C\;12:0+4\;C\;14:0+C\;16:0}{\mathrm{MUFA}+n-6+n-3}\;;$$

$$\mathrm{TI}=\frac{C\;14:0+C\;16:0+C\;18:0}{0.5\;\mathrm{MUFA}+0.5\;n-6+3\;n-3+\frac{n-3}{\begin{array}{c} n-6 \end{array}}}\;;$$

$$S/P=\frac{C\;14:0+C\;16:0+C\;18:0}{\mathrm{MUFA}+\mathrm{PUFA}};$$

$$\mathrm{NV}=\frac{C\;12:0+C\;14:0+C\;16:0}{C\;18:1+C\;18:2\;n-6}\;;$$

$$h/H=\frac{C\;18:1+C\;18:2\;n-6+\;C\;18:3\;n-3+C\;20:3\;n-6+C\;20:4\;n-6+\;C\;20:5\;n-3+\;C\;22:5\;n-3}{C\;12:0+C\;14:0+C\;16:0}$$

### 4.6. Statistical Analyses

Three-way variance analysis (ANOVA) was used to evaluate changes in the fatty acids profile occurring in the stored goose breast muscles. The effect of such factors as temperature (1 °C, 4 °C), the type of atmosphere (vacuum, modified atmosphere), and the storage time (four, seven, eleven days), as well as a couple of interactions between them were analyzed. When a significant main effect or interaction was identified, the mean values were further analyzed using the Tukey’s multiple range test. The results were presented as a mean ± standard deviation. Experimental data were analyzed using Statistica version 13.3 (Statsoft Inc., Kraków, Poland) [46]. Differences with a probability level < 0.05 were considered significant.

### 4.7. Ethical Statement

Ethical review and approval were waived for this study due to the research being based on a food product, goose meat, which is commercially available on the market.

## 5. Conclusions

From a nutritional point of view, the most favorable indicators, including the lowest level of saturation and the most favorable proportion of hypo- and hypercholesterolemic acids and the weakest athero- and thrombogenic effects, were found in the goose breast muscles stored in a vacuum at both 1 °C and 4 °C. At the same time, it was shown that in the goose meat stored in the high-oxygen modified atmosphere, irrespective of the storage temperature, the content of saturated fatty acids increased, in contrast to the polyunsaturated fatty acids, whose content decreased. This relationship is in contradiction to the current scientific recommendations, which stipulates the lowest possible proportion of saturated fatty acids in the diet and at the same time recommend changing the intake of saturated fatty acids in favor of polyunsaturated fatty acids.

## Figures and Tables

**Table 1:** The nutritional value of the breast muscles (without skin) of different species of poultry per 100 g of raw meat [7].

		Amount in 100 g of Raw Edible Portion			
Nutrients	Unit	Chicken	Turkey	Duck	Goose
Energy	kcal	120	114	123	133
Water	g	73.90	74.89	75.51	70.78
Protein	g	22.50	23.66	19.85	24.31
Fat	g	2.62	1.48	4.25	4.02
Sodium, Na	mg	45	113	57	50
Potassium, K	mg	334	242	268	336
Calcium, Ca	mg	5	11	3	4
Phosphorus, P	mg	213	201	186	256
Magnesium, Mg	mg	28	28	22	29
Iron, Fe	mg	0.37	0.73	4.51	5.91
Zinc, Zn	mg	0.68	1.28	0.74	1.68
Copper, Cu	mg	0.04	0.07	0.33	0.44
Manganese, Mn	mg	0.01	0.01	0.02	0.05
Vitamin A	µg	9	6	16	11
Vitamin E	mg	0.56	0.06	0	0.57
Thiamin	mg	0.09	0.04	0.42	0.28
Riboflavin	mg	0.18	0.15	0.31	1.52
Niacin	mg	9.60	9.92	3.44	6.56
Vitamin B_6_	mg	0.81	0.81	0.63	1.07
Vitamin B_12_	µg	0.21	0.70	0.63	4.10
SFA	g	0.56	0.29	1.32	0.61
MUFA	g	0.69	0.26	1.21	0.82
PUFA	g	0.424	0.258	0.58	0.35
Cholesterol	mg	73	57	77	80

**Table 2:** The results of the three-way variance analysis (ANOVA with interactions)—*p* value for the F test.

	Main Effect			Two-Way Interaction Effect			Three-Way Interaction Effect
Fatty Acid/ Dietary Index	Time	Atmosphere	Temperature	Time × Atmosphere	Time × Temperature	Atmosphere × Temperature	Time × Atmosphere × Temperature
SFA	0.000 ***	0.000 ***	0.000 ***	0.000 ***	0.000 ***	0.000 ***	0.000 ***
C 16:0	0.000 ***	0.000 ***	0.000 ***	0.000 ***	0.000 ***	0.000 ***	0.000 ***
MUFA	0.000 ***	0.000 ***	0.020 *	0.000 ***	0.532 ^ns^	0.003 **	0.051 ^ns^
C 18:1	0.000 ***	0.000 ***	0.592 ^ns^	0.000 ***	0.779 ^ns^	0.241 ^ns^	0.731 ^ns^
PUFA	0.000 ***	0.000 ***	0.000 ***	0.000 ***	0.000 ***	0.000 ***	0.000 ***
C 18:2 n-6	0.000 ***	0.000 ***	0.000 ***	0.000 ***	0.000 ***	0.000 ***	0.000 ***
C 18:3 n-3	0.000 ***	0.000 ***	0.022 *	0.000 ***	0.291 ^ns^	0.112 ^ns^	0.049 *
C 20:4 n-6	0.000 ***	0.000 ***	0.003 **	0.000 ***	0.016 *	0.004 **	0.196 ^ns^
PUFA/SFA	0.000 ***	0.000 ***	0.000 ***	0.000 ***	0.000 ***	0.000 ***	0.000 ***
UFA	0.000 ***	0.000 ***	0.141 ^ns^	0.000 ***	0.007 **	0.291 ^ns^	0.024 **
n-6	0.000 ***	0.000 ***	0.000 ***	0.000 ***	0.000 ***	0.000 ***	0.000 ***
n-3	0.000 ***	0.000 ***	0.014 *	0.000 ***	0.033 *	0.046 *	0.260 ^ns^
n-6/n-3	0.000 ***	0.000 ***	0.000 ***	0.000 ***	0.003 **	0.000 ***	0.001 ***
AI	0.000 ***	0.000 ***	0.000 ***	0.000 ***	0.000 ***	0.000 ***	0.000 ***
TI	0.000 ***	0.000 ***	0.000 ***	0.000 ***	0.000 ***	0.000 ***	0.000 ***
S/P	0.000 ***	0.000 ***	0.000 ***	0.000 ***	0.000 ***	0.000 ***	0.000 ***
NV	0.000 ***	0.000 ***	0.000 ***	0.000 ***	0.000 ***	0.000 ***	0.000 ***
h/H	0.000 ***	0.000 ***	0.000 ***	0.000 ***	0.000 ***	0.000 ***	0.000 ***

Significance effects: *** *p* < 0.001; ** 0.001 ≤ *p* < 0.01; * 0.01 ≤ *p* < 0.05; ns—not significant. SFA—saturated fatty acids, MUFA—monounsaturated fatty acids, PUFA—polyunsaturated fatty acids, UFA—unsaturated fatty acids, AI—atherogenic index, TI—thrombogenic index, S/P—saturation index, NV—lipid nutritional value, h/H—hypocholesterolemic/hypercholesterolemic ratio.

**Table 3:** The significance of the differences for mean values of fatty acid profile (% of the total fatty acids) and dietary indexes of goose meat stored in different atmospheres at 1 °C and 4 °C for up to 11 days in the post hoc Tukey’s test (*p* < 0.05).

Fatty Acids/Dietary Index	Atmosphere	Temperature	Storage Time (Days)			
			0	4	7	11
SFA	MA	1 °C	29.65 ^a^ ± 0.44	30.02 ^bx^ ± 0.18	31.07 ^cx^ ± 0.17	31.68 ^dx^ ± 0.23
		4 °C		30.15 ^bx^ ± 0.19	31.14 ^cx^ ± 0.16	33.12 ^dy^ ± 0.19
	Vacuum	1 °C		29.66 ^ay^ ± 0.21	29.65 ^ay^ ± 0.16	29.75 ^az^ ± 0.14
		4 °C		29.58 ^ay^ ± 0.19	29.69 ^ay^ ± 0.14	29.65 ^az^ ± 0.21
C 16:0	MA	1 °C	24.04 ^a^ ± 0.38	24.09 ^ax^ ± 0.19	24.86 ^bx^ ± 0.13	24.94 ^bx^ ± 0.16
		4 °C		24.13 ^ax^ ± 0.20	24.87 ^bx^ ± 0.12	25.99 ^cy^ ± 0.13
	Vacuum	1 °C		24.01 ^ax^ ± 0.13	24.02 ^ay^ ± 0.15	24.06 ^az^ ± 0.15
		4 °C		23.96 ^ax^ ± 0.14	23.98 ^ay^ ± 0.12	23.96 ^az^ ± 0.15
MUFA	MA	1 °C	42.88 ^a^ ± 0.36	43.84 ^bx^ ± 0.51	44.22 ^bx^ ± 0.30	45.10 ^cx^ ± 0.39
		4 °C		44.11 ^bx^ ± 0.25	44.38 ^bx^ ± 0.24	45.55 ^cy^ ± 0.31
	Vacuum	1 °C		42.87 ^ay^ ± 0.36	42.98 ^ay^ ± 0.32	43.04 ^az^ ± 0.35
		4 °C		42.91 ^ay^ ± 0.49	42.90 ^ay^ ± 0.58	42.96 ^az^ ± 0.26
C 18:1	MA	1 °C	38.78 ^a^ ± 0.31	39.88 ^bx^ ± 0.40	39.98 ^cx^ ± 0.22	40.65 ^dx^ ± 0.34
		4 °C		39.93 ^bx^ ± 0.30	40.00 ^cx^ ± 0.23	40.84 ^dx^ ± 0.24
	Vacuum	1 °C		38.86 ^ax^ ± 0.52	39.03 ^ay^ ± 0.53	38.88 ^ay^ ± 0.39
		4 °C		38.79 ^ax^ ± 0.47	39.03 ^ay^ ± 0.56	38.86 ^ay^ ± 0.28
PUFA	MA	1 °C	25.89 ^a^ ± 0.27	24.72 ^bx^ ± 0.40	23.35 ^cx^ ± 0.12	21.35 ^dx^ ± 0.12
		4 °C		24.66 ^bx^ ± 0.43	23.19 ^cx^ ± 0.22	20.11 ^dy^ ± 0.40
	Vacuum	1 °C		25.95 ^ay^ ± 1.32	25.81 ^ay^ ± 0.26	25.88 ^az^ ± 0.14
		4 °C		25.87 ^ay^ ± 0.30	25.80 ^ay^ ± 0.28	25.98 ^az^ ± 0.14
C 18:2 n-6	MA	1 °C	18.92 ^a^ ± 0.10	18.00 ^bx^ ± 0.13	17.50 ^cx^ ± 0.14	16.26 ^dx^ ± 0.12
		4 °C		18.02 ^bx^ ± 0.16	17.47 ^cx^ ± 0.16	15.18 ^dy^ ± 0.26
	Vacuum	1 °C		18.89 ^ay^ ± 0.12	18.80 ^ay^ ± 0.12	18.87 ^az^ ± 0.14
		4 °C		18.87 ^ay^ ± 0.13	18.76 ^ay^ ± 0.13	18.88 ^az^ ± 0.13
C 18:3 n-3	MA	1 °C	1.76 ^a^ ± 0.07	1.51 ^bx^ ± 0.03	1.44 ^cx^ ± 0.09	1.31 ^dx^ ± 0.05
		4 °C		1.58 ^by^ ± 0.04	1.48 ^cx^ ± 0.05	1.30 ^dx^ ± 0.08
	Vacuum	1 °C		1.79 ^az^ ± 0.05	1.77 ^ay^ ± 0.07	1.77 ^ay^ ± 0.05
		4 °C		1.78 ^az^ ± 0.02	1.78 ^ay^ ± 0.05	1.79 ^ay^ ± 0.06
C 20:4 n-6	MA	1 °C	4.45 ^a^ ± 0.23	4.54 ^ax^ ± 0.24	3.86 ^bx^ ± 0.08	3.31 ^cx^ ± 0.10
		4 °C		4.39 ^ax^ ± 0.26	3.68 ^by^ ± 0.08	3.21 ^cx^ ± 0.08
	Vacuum	1 °C		4.52 ^ax^ ± 0.18	4.56 ^az^ ± 0.17	4.46 ^ay^ ± 0.19
		4 °C		4.45 ^ax^ ± 0.16	4.50 ^az^ ± 0.21	4.58 ^ay^ ± 0.08
PUFA/SFA	MA	1 °C	0.87 ^a^ ± 0.02	0.82 ^bx^ ± 0.01	0.75 ^bx^ ± 0.00	0.67 ^cx^ ± 0.00
		4 °C		0.82 ^bx^ ± 0.01	0.74 ^cx^ ± 0.01	0.61 ^dy^ ± 0.01
	Vacuum	1 °C		0.88 ^ay^ ± 0.02	0.87 ^ay^ ± 0.01	0.87 ^az^ ± 0.00
		4 °C		0.87 ^ay^ ± 0.01	0.87 ^ay^ ± 0.01	0.88 ^az^ ± 0.00
UFA	MA	1 °C	68.77 ^a^ ± 0.6	68.57 ^ax^ ± 0.64	67.57 ^bx^ ± 0.39	66.45 ^cx^ ± 0.49
		4 °C		68.78 ^ax^ ± 0.58	67.56 ^ax^ ± 0.37	65.66 ^cy^ ± 0.57
	Vacuum	1 °C		68.82 ^ax^ ± 0.80	68.99 ^ay^ ± 0.63	69.02 ^az^ ± 0.39
		4 °C		68.79 ^ax^ ± 0.71	69.01 ^ay^ ± 0.62	68.94 ^az^ ± 0.35
n-6	MA	1 °C	23.72 ^a^ ± 0.23	22.86 ^bx^ ± 0.35	21.67 ^cx^ ± 0.12	19.85 ^dx^ ± 0.11
		4 °C		22.73 ^bx^ ± 0.39	21.44 ^cx^ ± 0.20	18.61 ^dy^ ± 0.32
	Vacuum	1 °C		23.77 ^ay^ ± 0.25	23.71 ^ay^ ± 0.19	23.70 ^az^ ± 0.16
		4 °C		23.69 ^ay^ ± 0.25	23.60 ^ay^ ± 0.25	23.81 ^az^ ± 0.12
n-3	MA	1 °C	2.17 ^a^ ± 0.06	1.86 ^bx^ ± 0.05	1.69 ^cx^ ± 0.10	1.50 ^dx^ ± 0.06
		4 °C		1.93 ^by^ ± 0.04	1.74 ^cx^ ± 0.07	1.50 ^dx^ ± 0.09
	Vacuum	1 °C		2.18 ^az^ ± 0.11	2.18 ^ay^ ± 0.05	2.18 ^ay^ ± 0.05
		4 °C		2.18 ^az^ ± 0.05	2.20 ^ay^ ± 0.06	2.17 ^ay^ ± 0.06
n-6/n-3	MA	1 °C	10.93 ^a^ ± 0.22	12.31 ^bx^ ± 0.17	12.90 ^cx^ ± 0.76	13.24 ^dx^ ± 0.55
		4 °C		11.80 ^by^ ± 0.17	12.31 ^cy^ ± 0.45	12.47 ^cy^ ± 0.53
	Vacuum	1 °C		10.94 ^az^ ± 0.50	10.90 ^az^ ± 0.39	10.87 ^az^ ± 0.27
		4 °C		10.86 ^az^ ± 0.14	10.77 ^az^ ± 0.26	10.96 ^az^ ± 0.29
AI	MA	1 °C	0.40 ^a^ ± 0.01	0.41 ^bx^ ± 0.00	0.43 ^cx^ ± 0.00	0.44 ^dx^ ± 0.01
		4 °C		0.41 ^bx^ ± 0.00	0.43 ^cx^ ± 0.00	0.46 ^dy^ ± 0.00
	Vacuum	1 °C		0.40 ^ax^ ± 0.00	0.40 ^ay^ ± 0.00	0.40 ^az^ ± 0.00
		4 °C		0.40 ^ax^ ± 0.00	0.40 ^ay^ ± 0.00	0.40 ^az^ ± 0.00
TI	MA	1 °C	0.73 ^a^ ± 0.01	0.76 ^bx^ ± 0.01	0.80 ^cx^ ± 0.01	0.83 ^dx^ ± 0.01
		4 °C		0.75 ^bx^ ± 0.00	0.80 ^cx^ ± 0.01	0.88 ^dy^ ± 0.01
	Vacuum	1 °C		0.73 ^ay^ ± 0.01	0.72 ^ay^ ± 0.00	0.73 ^az^ ± 0.00
		4 °C		0.73 ^ay^ ± 0.01	0.72 ^ay^ ± 0.00	0.73 ^az^ ± 0.00
S/P	MA	1 °C	0.42 ^a^ ± 0.01	0.43 ^bx^ ± 0.00	0.45 ^cx^ ± 0.00	0.46 ^dx^ ± 0.00
		4 °C		0.43 ^bx^ ± 0.00	0.45 ^cx^ ± 0.00	0.49 ^dy^ ± 0.00
	Vacuum	1 °C		0.42 ^ay^ ± 0.00	0.42 ^ay^ ± 0.00	0.42 ^az^ ± 0.00
		4 °C		0.42 ^ay^ ± 0.00	0.42 ^ay^ ± 0.00	0.42 ^az^ ± 0.00
NV	MA	1 °C	0.43 ^a^ ± 0.00	0.43 ^ax^ ± 0.00	0.45 ^bx^ ± 0.00	0.46 ^cx^ ± 0.00
		4 °C		0.44 ^ax^ ± 0.00	0.45 ^bx^ ± 0.00	0.49 ^cy^ ± 0.00
	Vacuum	1 °C		0.43 ^ax^ ± 0.00	0.43 ^ay^ ± 0.01	0.43 ^az^ ± 0.00
		4 °C		0.43 ^ax^ ± 0.00	0.43 ^ay^ ± 0.00	0.43 ^az^ ± 0.00
h/H	MA	1 °C	2.58 ^a^ ± 0.04	2.56 ^ax^ ± 0.02	2.43 ^bx^ ± 0.01	2.37 ^cx^ ± 0.01
		4 °C		2.56 ^ax^ ± 0.02	2.42 ^bx^ ± 0.01	2.24 ^cy^ ± 0.02
	Vacuum	1 °C		2.59 ^ax^ ± 0.02	2.59 ^ay^ ± 0.03	2.58 ^az^ ± 0.02
		4 °C		2.58 ^ax^ ± 0.02	2.59 ^ay^ ± 0.03	2.59 ^az^ ± 0.01

The data are average values of 25 tests for storage time 0, 4, 7, 9, 11 days ± standard deviation. a–d: Means with different letters in the same row differ at *p* ˂ 0.05 in view of the time of storage. x–z: Means with different letters in the same column differ at *p* ˂ 0.05 in view of the packaging atmosphere and temperature. Significance effects: * *p* ˂ 0.05. MA: modified atmosphere, 80% O_2_ and 20% CO_2_. SFA—saturated fatty acids, MUFA—monounsaturated fatty acids, PUFA—polyunsaturated fatty acids, UFA—unsaturated fatty acids, AI—atherogenic index, TI—thrombogenic index, S/P—saturation index, NV—lipid nutritional value, h/H—hypocholesterolemic/hypercholesterolemic ratio.

## Data Availability

Not applicable.

## Sample Availability

Samples of the compounds are not available from the authors.

## References

[1] Tavoletti S., Foligni R., Mozzon M., Pasquini M. (2018). Comparison between fatty acid profiles of old and modern varieties of *T. turgidum* and *T. aestivum*: A case study in central Italy. J. Cereal Sci..

[2] Ruschioni S., Loreto N., Foligni R., Mannozzi C., Raffaelli N., Zamporlini F., Pasquini M., Roncolini A., Cardinali F., Osimani A. (2020). Addition of Olive Pomace to Feeding Substrate Affects Growth Performance and Nutritional Value of Mealworm (*Tenebrio molitor* L.) Larvae. Foods.

[3] Tavoletti S., Pasquini M., Mozzon M., Servadio D., Merletti A., Mannozzi C., Foligni R. (2021). Discrimination among varieties of *Triticum turgidum* subspecies (*dicoccon, turanicum* and *durum*) based on the fatty acid profile. J. Cereal Sci..

[4] Maoloni A., Milanović V., Osimani A., Cardinali F., Garofalo C., Foligni R., Mozzon M., Cirlini M., Spaggiari M., Reale A. (2021). Exploitation of sea fennel (*Crithmum maritimum* L.) for manufacturing of novel high-value fermented preserves. Food Bioprod. Process..

[5] OECD/FAO (2021). OECD-FAO Agricultural Outlook 2021–2030.

[6] OECD/FAO (2020). OECD-FAO Agricultural Outlook 2020–2029.

[7] United States Department of Agriculture Agricultural Research Service. www.ars.usda.gov/ARSUserFiles/80400535/Data/SR/SR28/reports/sr28fg05.pdf.

[8] Buzala M., Adamski A., Janicki B. (2014). Characteristics of performance traits and the quality of meat and fat in Polish oat geese. World’s Poult. Sci. J..

[9] Orkusz A. (2021). Edible insects versus meat—Nutritional comparison: Knowledge of their composition is the key to good health. Nutrients.

[10] Simopoulos A.P. (2016). An Increase in the Omega-6/Omega-3 Fatty Acid Ratio Increases the Risk for Obesity. Nutrients.

[11] Amaral A.B., da Silva M.V., da Silva Lannes S.C. (2018). Lipid oxidation in meat: Mechanisms and protective factors—A review. Food Sci. Technol..

[12] Lawrence G.D. (2010). The Fats of Life: Essential Fatty Acids in Health and Disease.

[13] Shramko V.S., Polonskaya Y.V., Kashtanova E.V., Stakhneva E.M., Ragino Y.I. (2020). The Short Overview on the Relevance of Fatty Acids for Human Cardiovascular Disorders. Biomolecules.

[14] Orkusz A., Haraf G., Okruszek A., Wereńska-Sudnik M. (2017). Lipid oxidation and color changes of goose meat stored under vacuum and modified atmosphere conditions. Poult. Sci..

[15] Ling T. (2015). Oxidation of Polyunsaturated Fatty Acids and its Impact on Food Quality and Human Health. Adv. Food Technol. Nutr. Sci. Open J..

[16] Honikel K.O., Toldrá F. (2009). Oxidative Changes and Their Control in Meat and Meat Products. Safety of Meat and Processed Meat. Food Microbiology and Food Safety.

[17] Popowa T. (2014). Fatty acid composition of Longissimus dorsi and Semimembranosus muscles during storage in lambs reared indoors and on pasture. Emir. J. Food Agric..

[18] Biesiada-Drzazga B. (2006). Description of selected characteristics of muscle and fat tissue of 10-week White Koluda W31^®^ geese. Acta Sci. Pol. Technol. Aliment..

[19] Gumułka M., Połtowicz K. (2020). Comparison of carcass traits and meat quality of intensively reared geese from a Polish genetic resource flock to those of commercial hybrids. Poult. Sci..

[20] Gumułka M., Kapkowska E., Borowiec F., Rabsztyn A., Połtowicz K. (2006). Fatty acid profile and chemical composition of muscles and abdominal fat in geese from genetic reserve and commercial flock. Anim. Sci..

[21] Meng H., Matthan N.R., Wu D., Li L., Rodríguez-Morató J., Cohen R., Galluccio J.M., Dolnikowski G.G., Lichtenstein A.H. (2019). Comparison of diets enriched in stearic, oleic, and palmitic acids on inflammation, immune response, cardiometabolic risk factors, and fecal bile acid concentrations in mildly hypercholesterolemic postmenopausal women—Randomized crossover trial. Am. J. Clin. Nutr..

[22] Shen H., Eguchi K., Kono N., Fujiu K., Matsumoto S., Shibata M., Oishi-Tanaka Y., Komuro I., Arai H., Nagai R. (2013). Saturated Fatty Acid Palmitate Aggravates Neointima Formation by Promoting Smooth Muscle Phenotypic Modulation Significance. Arter. Thromb. Vasc. Boil..

[23] Sacks F.M., Lichtenstein A.H., Wu J.H., Appel L.J., Creager M.A., Kris-Etherton P.M., Miller M., Rimm E.B., Rudel L.L., Robinson J.G. (2017). Dietary Fats and Cardiovascular Disease: A Presidential Advisory From the American Heart Association. Circulation.

[24] Chiuve S.E., Rimm E.B., Sandhu R.K., Bernstein A.M., Rexrode K.M., Manson J.E., Willett W.C., Albert C.M. (2012). Dietary fat quality and risk of sudden cardiac death in women. Am. J. Clin. Nutr..

[25] Food and Agriculture Organization of the United Nations/World Health Organization/United Nations University (FAO/WHO/UNU) (2004). Human Energy Requirements, Report of a Joint FAO/WHO/UNU Expert Consultation.

[26] EFSA Panel on Dietetic Products, Nutrition and Allergies (NDA) (2010). Scientific Opinion on Dietary Reference Values for fats, including saturated fatty acids, polyunsaturated fatty acids, monounsaturated fatty acids, trans fatty acids, and cholesterol. EFSA J..

[27] Eckel R.H., Jakicic J.M., Ard J.D., De Jesus J.M., Hubbard V.S., Lee I.-M., Lichtenstein A.H., Loria C.M., Millen B.E., Nonas C.A. (2013). 2013 AHA/ACC Guideline on Lifestyle Management to Reduce Cardiovascular Risk: A Report of the American College of Cardiology/American Heart Association Task Force on Practice Guidelines. Circulation.

[28] WHO (2018). Draft Guidelines on Saturated Fatty Acid and Trans-Fatty Acid Intake for Adults and Children. https://extranet.who.int/dataform/upload/surveys/666752/files/Draft%20WHO%20SFA-TFA%20guidelines_04052018%20Public%20Consultation(1).pdf.

[29] Arnett D.K., Blumenthal R.S., Albert M.A., Buroker A.B., Goldberger Z.D., Hahn E.J., Himmelfarb C.D., Khera A., Lloyd-Jones D., McEvoy J.W. (2019). 2019 ACC/AHA Guideline on the Primary Prevention of Cardiovascular Disease: Executive Summary: A Report of the American College of Cardiology/American Heart Association Task Force on Clinical Practice Guideline. J. Am. Coll. Cardiol..

[30] Hlais S., El-Bistami D., El Rahi B., Mattar M.A., Obeid O. (2013). Combined Fish Oil and High Oleic Sunflower Oil Supplements Neutralize their Individual Effects on the Lipid Profile of Healthy Men. Lipids.

[31] Pérez-Martínez P., García-Ríos A., Delgado F.G., Jiménez F.P., López-Miranda J. (2011). Mediterranean diet rich in olive oil and obesity, metabolic syndrome and diabetes mellitus. Curr. Pharm. Des..

[32] Wasowicz E., Gramza A., Hes M., Jelen H.H., Korczak J., Malecka M., Mildner-Szkudlarz S., Rudzinska M., Samotyja U., Zawirska-Wojtasiak R. (2004). Oxidation of lipids in food. Pol. J. Food Nutr. Sci..

[33] Forouhi N.G., Krauss R.M., Taubes G., Willet W. (2018). Dietary fat and cardiometabolic health: Evidence, controversies, and consensus for guidance. BMJ.

[34] Bentsen H. (2017). Dietary polyunsaturated fatty acids, brain function and mental health. Microb. Ecol. Health Dis..

[35] Layé S., Nadjar A., Joffre C., Bazinet R.P. (2018). Anti-Inflammatory Effects of Omega-3 Fatty Acids in the Brain: Physiological Mechanisms and Relevance to Pharmacology. Pharmacol. Rev..

[36] Du Y., Taylor C.G., Zahradka P. (2019). Modulation of endothelial cell responses and vascular function by dietary fatty acids. Nutr. Rev..

[37] Liu Y., Poon S., Seeman E., Hare D.L., Bui M., Iuliano S. (2019). Fat from dairy foods and ‘meat’ consumed within recommended levels is associated with favourable serum cholesterol levels in institutionalised older adults. J. Nutr. Sci..

[38] Ratnayake W.M.N., Galli C. (2009). Fat and Fatty Acid Terminology, Methods of Analysis and Fat Digestion and Metabolism: A Background Review Paper. Ann. Nutr. Metab..

[39] (2007). Food, Nutrition, Physical Activity and the Prevention of Cancer: A Global Perspective.

[40] Czechlowska T., Bielińska H. (1993). Technology of Rearing and Fattening of White Kołuda Geese.

[41] Folch J., Less M., Sloane J., Stanley G.H. (1956). A simple method for the isolation and purification of total lipids from animal tissues. J. Biol. Chem..

[42] Ulbricht T.L.V., Southgate D.A.T. (1991). Coronary disease seven dietary factors. Lancet.

[43] Estévez M., Morcuende D., Ramírez R., Ventanas S., Cava R. (2004). Extensively reared Iberian pigs versus intensively reared white pigs for the manufacture of liver pâté. Meat Sci..

[44] Fernandez M., Ordonez J.A., Cambero I., Santos C., Pin C., De la Hoz L. (2007). Fatty acid compositions of selected varieties of Spanish dry ham related to their nutritional implications. Food Chem..

[45] Santos-Silva J., Bessa R.J.B., Santos-Silva F. (2002). Effect of genotype, feeding system and slaughter weight on the quality of light lambs. II. Fatty acid composition of meat. Livest. Prod. Sci..

[46] (2013). Statistica, Version 13.3.

